# Non-preventable cases of breast, prostate, lung, and colorectal cancer in 2050 in an elimination scenario of modifiable risk factors

**DOI:** 10.1038/s41598-024-59314-x

**Published:** 2024-04-13

**Authors:** Frederik Knude Palshof, Lina Steinrud Mørch, Brian Køster, Gerda Engholm, Hans Henrik Storm, Therese M.-L. Andersson, Niels Kroman

**Affiliations:** 1 Danish Cancer Institute, Danish Cancer Society, Copenhagen, Denmark; 2 Department of Breast Surgery, Gentofte Hospital, Hellerup, Denmark; 3 Department of Medical Epidemiology and Biostatistics, Karolinska Institutet, Stockholm, Sweden

**Keywords:** Breast cancer, Cancer prevention, Gastrointestinal cancer, Lung cancer, Urological cancer, Risk factors, Disease prevention, Epidemiology

## Abstract

Most Western countries have increasing number of new cancer cases per year. Cancer incidence is primarily influenced by basically avoidable risk factors and an aging population. Through hypothetical elimination scenarios of multiple major risk factors for cancer, we estimated the number of new cancer cases that are non-preventable in 2050. We compare numbers of new postmenopausal breast, prostate, lung, and colorectal cancer cases in 2021 to projected numbers of new cases in 2050 under prevention scenarios regarding smoking, overweight and obesity, and alcohol consumption: no intervention, 50%, and 100% instant reduction. Cancer incidence data were derived from NORDCAN, and risk factor prevalence data from the Danish National Health Survey. Cancer projections were calculated with the Prevent program. Hypothetical 100% instant elimination of major risk factors for cancer in Denmark in 2022 will result in unchanged numbers of new breast and colorectal cancers in 2050. The number of new prostate cancers will increase by 25% compared to 2021. Unchanged risk factor levels will result in noticeable increase in cancer burden. Increase in life expectancy and age will entail an increase in cancer incidence, despite maximum effect of preventive actions in the population. Our results are important when planning future health care.

## Introduction

In recent years, the number of new cancer cases per year has risen in Western countries^1^, dominated by the most common cancers, breast, prostate, lung, and colorectal cancer. Data on incident cancers in the Nordic countries can be found in the Nordic cancer statistics database NORDCAN^2,3^. This is based on the Nordic cancer registries and thus has a high level of completeness. Prediction estimates made in NORDCAN for the Nordic countries for these common cancers suggest that during the next 20 years, the number of new cases will, on average, increase by 26%. The most significant increases for colorectal cancer (29%) and prostate cancer (26.5%)^4,5^^.^

A larger number of cancer cases will increase the pressure on the healthcare system. Preventive actions not accounted for in NORDCAN could be taken, changing the projections for cancer in the Nordic populations.

Modifiable risk factors that increase the risk of the most common cancers are widely described and documented, especially when it comes to tobacco smoking^6–8^, overweight and obesity^9–11^, and alcohol consumption^12–16^. Reducing the prevalence of these factors in a population is thus a general approach for preventing future cancer cases. Studies on the number of incident cancers that could be avoided in the Nordic countries by various prevention strategies are described by Andersson et al.^17–19^. These studies state that over 30 years, 133,000 breast, lung, and colorectal cancer cases could be avoided, equivalent to about 15% of incident cancers related to the mentioned risk factors. However, these studies were based on calculations for one risk factor at a time rather than combinations of multiple risk factors. A study by Islami et al.^20^ reported that 32.4% of all cancer incidents in the U.S. in 2014 were caused by tobacco smoking, excess body weight, or alcohol consumption.

Age, however, is the most critical risk factor for developing cancer, especially in developing epithelial cancers, where increasing age dramatically increases the risk^21,22^. In a Western country like Denmark, the number of people aged 65 years and older will increase by 34%, and the number of people older than 80 years will more than double from 2020 to 2050, according to Statistics Denmark^23^. Presently, in Denmark, 66% of cancer cases are diagnosed in individuals aged 65 years and above^24,25^.

As current predictions show a future with more individuals getting a cancer diagnosis, knowledge is needed about the number of non-preventable cancers in scenarios of prevention strategies targeting multiple major risk factors related to cancer. This could aid in future health planning strategies.

The present study’s purpose was to estimate the number of new cases of postmenopausal breast, prostate, lung, and colorectal cancer in 2050 in Denmark compared to 2021 in three prevention scenarios regarding smoking, overweight and obesity, and alcohol consumption. The prevention scenarios will imply no, half, or total elimination of smoking, overweight and obesity, and alcohol consumption beginning in 2022. Our study is based on Danish data, and our methods and results can largely be applied to most other Western countries.

## Methods

We estimated the number of new cancer cases in the year 2050 for the four most common cancers (breast, prostate, lung, and colorectal cancer) in Denmark under different prevalence scenarios of tobacco smoking, overweight and obesity, and alcohol consumption. We compared the number of new cancer cases in 2050 to the number of cancer cases in Denmark in 2021 under the different prevalence scenarios to evaluate the potential effects of prevention seen from today’s perspective.

We used the macrosimulation model Prevent, a program developed to model prevention scenarios. In summary, the model estimates disease incidence in future years based on different scenarios in the prevalence of risk factors. It is based on data from a starting year, such as cancer incidence rates, population size, and age distribution. The model simulates changes in specific age groups, and in our study, we used Prevent to estimate changes in future cancer incidence. The model was adapted for the EUROCADET project^26–28^ and is, in general, described in papers by Gunningschepers et al.^29^ and Soerjomataram et al.^30^;

Prevent requires input data in the form of disease incidence, risk factor prevalence (historical and current), population size (including future projections), relative risk (RR) estimates, and the changes in prevalence of risk factors in the different projected scenarios. All these inputs can vary by age and sex.

When estimating the preventive effect of multiple risk factors for one disease, Prevent assumes that relative risks are multiplicative and that the risk factors are distributed independently in the population.

### Disease incidence

Incidence rates of female postmenopausal breast cancer (International Classification of Diseases 10th revision (ICD10): C50), prostate cancer (ICD10: C61), lung cancer (ICD10: C33-3C4), and colon (ICD10: C18) and rectal cancer (ICD10: C19-C20) were retrieved from the Nordic cancer statistics database NORDCAN^24,25^. For postmenopausal breast cancer, only the incidence in women from 50 years of age was considered.

To avoid variation in the data due to late registration and the COVID-19 pandemic, we used the average incidence for 2018–2021. The incidence was included for 5-year age groups (15–19, 20–24, …, 80–84, 85+).

### Disease trend

For lung cancer, we applied a disease trend based on the estimated annual percentage change (EAPC) in the period from 1996 to 2021 from NORDCAN using the NORDPRED-model^31^ implemented in the database. We calculated the mean EAPC and extended the trend to 2050, which was applied to 2022 to 2050 in our model.

For breast, prostate, and colorectal cancer, we used the constant rate 2018–2021, with no change in disease trend. This was due to the implementation of screening for breast and colorectal cancer in 2009 and 2014, respectively, and the excessive increase in prostate-specific antigen (PSA) use in Denmark.

### Risk factors and risk estimates

Risk factors for the four cancer types were included based on the classification by the International Agency for Research on Cancer (IARC) as “Carcinogenic agents with *sufficient evidence* in humans”^32^ or by the World Cancer Research Fund (WCRF) as “convincing evidence” as a cause of the specific cancer^33–36^. For breast cancer, overweight and obesity, and alcohol consumption were considered. For colon and rectal cancer, smoking, overweight and obesity, and alcohol consumption were considered, and for lung cancer, smoking was considered the only factor. No preventable risk factors matched the criteria for prostate cancer. We did not consider risk factors with protective effects on cancer. Tobacco smoking status was categorized as never smoker, secondhand smoker (never-smokers exposed to smoking 30 min a day), former smoker (used to be smoking but quit), or current smoker (daily smoker). Occasional smokers were considered never smokers. The RR estimates used were based on findings from Gandini^37^ and Kim^38^, also used in a study by Andersson in 2018^18^ and Tybjerg et al. in 2022^39^ (Table 1).

**Table 1:** Cancer type and relative risk estimates (RR) in different exposure categories for tobacco smoking status, BMI, and alcohol consumption in Denmark.

Risk factor	Cancer type	Relative risk estimates			
Tobacco smoking		Never/occasional smoker	Secondhand smoker	Former smoker	Current smoker
	Lung	1.0 (Ref)	(F^a^) 1.37 (1.23–1.52) (M^b^) 1.23 (1.10–1.38)	3.85 (2.77–5.34)	8.96 (6.63–12.11)
	Colon	1.0 (Ref)	1.0	1.15 (1.07–1.25)	1.11 (1.00–1.24)
	Rectal	1.0 (Ref)	1.0	1.11 (1.02–1.20)	1.44 (1.18–1.77)
BMI		Healthy weight	Overweight	Obesity	
	Breast	1.0 (Ref)	1.13 (1.09–1.18)	1.20 (1.11–1.31)	
	Colorectal	1.0 (Ref)	(F) 1.07 (1.01–1.14) (M) 1.17 (1.12–1.22)	(F) 1.17 (1.06–1.30) (M) 1.38 (1.32–1.44)	
Alcohol consumption		Non/occasional drinker	Light drinker	Medium drinker	Heavy drinker
	Breast	1.0 (Ref)	1.04 (1.01–1.07)	1.23 (1.19–1.28)	1.61 (1.33–1.94)
	Colorectal	1.0 (Ref)	0.99 (0.95–1.04)*	1.17 (1.11–1.24)	1.44 (1.25–1.65)

A 95% confidence interval is shown in parentheses.

*RR is assumed to be 1 for the calculations.

^a^Female.

^b^Male.

Overweight and obesity were defined by Body Mass Index (BMI) (weight (in kg)/height (in m)^2^). The groups were categorized according to the World Health Organization (WHO) criteria^40^: healthy weight BMI < 25 (underweight was considered as healthy weight), overweight 25 ≤ BMI < 30, and obesity BMI ≥ 30. We used the same RR estimates for overweight and obese individuals compared to healthy-weight individuals, as reported by Xue^41^ and Munsell^42^ and WCRF^33^ (Table 1).

Alcohol consumption in the population was categorized as non/occasional drinkers, light drinkers (≤ 1 drink per day), moderate drinkers (> 1 and ≤ 4 drinks per day), or heavy drinkers (> 4 drinks per day), assuming 12.5 g of alcohol (ethanol) to be equal to one drink. The RR estimates used were based on findings from Bagnardi et al.^16^, also used in studies by Andersson et al.^17^ and Tybjerg et al*.*^39^ (Table 1). Due to different estimates for colon and rectal cancer, estimates were calculated independently for the two types of cancer and then added together in the results for *colorectal cancer*.

### Estimated population size

Demographic data on Denmark's estimated future population size were collected from population projections in Statistics Denmark^43^ by sex and 5-year age groups (0–4, 5–9…,80–84, 85+). The base year was 2021, and estimates were used for 2023–2050. For 2021 and 2022, actual numbers were utilized according to sex and 1-year age groups (0, 1,…, 84, 85+).

### Risk factor prevalence

Prevalence data on tobacco smoking status, BMI, and alcohol consumption were collected from The Danish National Health Survey^44^ by sex and 10-year age groups (16–24, 25–34 … 75+) from the most recent years available (2010, 2013, 2017 and 2021). The prevalence of risk factors was treated as categorical. Data on children under 16 were not obtainable and, therefore, excluded.

### Disease latency and lag years

When individuals are no longer exposed to a risk factor, their risk of disease decreases with time towards that of unexposed individuals, e.g., when quitting smoking, the risk of lung cancer is unchanged for a period until it starts declining and levels with individuals who are never smokers. The Prevent model considers this as latency time and lag years. Latency time was defined as the time, in years, from a change in exposure to a risk factor until the risk of disease started to change. Lag years were defined as the number of years from the onset of the transition in disease risk to the point at which the disease risk among previously exposed individuals aligns with the risk observed in the unexposed population. Assumptions for each disease and risk factor can be found in the supplementary material (Supplementary A).

### Intervention scenarios

We assumed the intervention would start in 2022 and continue until 2050. When the prevalence was lowered, the age groups were allocated to the reference risk estimates (data shown in Supplementary A).

A: Half reduction.

This implies a 50% reduction in the prevalence of secondhand, former, and current smokers, overweight and obese, and light, medium, and heavy drinkers from 2022.

B: Full elimination.

This implies a total reduction in the prevalence of secondhand, former, and current smokers, overweight and obese, and light, medium, and heavy drinkers from 2022.

We performed sensitivity analyses in all scenarios using the risk estimates' lowest and highest 95% confidence intervals, presented in the supplementary material (Supplementary B). We also compared the no-intervention scenario predictions with the NORDCAN predictions, shown in the supplementary material (Supplementary A).

## Results

Tables 2 and 3 present the estimated number of new cases of prostate, female postmenopausal breast, colorectal, and lung cancer in Denmark in 2050 under the three scenarios regarding modifiable risk factors: without intervention, scenario A (50% instant reduction), and scenario B (100% instant elimination).

**Table 2:** Non-preventable cancer cases in 2050 in Denmark predicted in numbers (#) and percentage with the current trend (No intervention) and in scenarios of reduction of modifiable risk factors.

Cancer type	No intervention	50% instant reduction*		100% instant elimination§	
	#	Intervention #	Difference¤ # (%)	Intervention #	Difference¤ # (%)
Prostate	5,775	5,775	0 (0)	5,775	0 (0)
Breast (postmenopausal)^a^	4,976	4,606	− 370 (− 7.4)	4,250	− 726 (− 14.6)
Colorectal, men^b^	3,484	2,977	− 507 (− 14.6)	2,521	− 963 (− 27.6)
Colorectal, women^b^	2,897	2,662	− 235 (− 8.1)	2,440	− 457 (− 15.8)
Lung, men^c^	2,085	1,339	− 746 (− 35.8)	593	− 1,492 (− 71.6)
Lung, women^c^	2,197	1,447	− 750 (− 34.1)	696	− 1,501 (− 68.3)
Lung, men and women^c^	4,282	2,786	− 1,496 (− 34.9)	1,289	− 2,993 (− 69.9)

*50% instant reduction in modifiable risk factors from 2022.

^§^100% instant elimination in modifiable risk factors from 2022.

¤Difference compared to no intervention scenario.

^a^Reduction in overweight and obesity, and alcohol consumption.

^b^Reduction in smoking, overweight and obesity, and alcohol consumption.

^c^Reduction in smoking.

**Table 3:** Non-preventable cancer cases in 2050 in Denmark predicted in numbers (#) and percentage, compared to the number of new cancer cases in 2021.

Cancer type	Year 2021	No intervention*		100% instant elimination§	
	#	Year 2050 #	Difference¤ # (%)	Year 2050 #	Difference¤ # (%)
Prostate	4,620	5,775	1,155 (25.0)	5,775	1,155 (25.0)
Breast (postmenopausal)^a^	4,172	4,976	804 (19.3)	4,250	78 (1.9)
Colorectal, men^b^	2,516	3,484	968 (38.5)	2,521	5 (0.2)
Colorectal, women^b^	2,171	2,897	726 (33.4)	2,440	269 (12.4)
Lung, men^c^	2,508	2,085	− 423 (− 16.9)	593	− 1,915 (− 76.4)
Lung, women^c^	2,693	2,197	− 496 (− 18.4)	696	− 1,997 (− 74.2)
Lung, women and men^c^	5,201	4,282	− 919 (− 17.7)	1,289	− 3,912 (− 75.2)

*No intervention: predicted cancer development based on current trends in cancer incidence.

^§^100% instant elimination: Full reduction in smoking, overweight and obesity, and alcohol consumption from 2022.

¤Estimated difference in new cancer cases in Denmark between 2050 and 2021.

^a^Reduction in overweight and obesity, and alcohol consumption.

^b^Reduction in smoking, overweight and obesity, and alcohol consumption.

^c^Reduction in smoking.

### Projected trend

**Figure 1:**
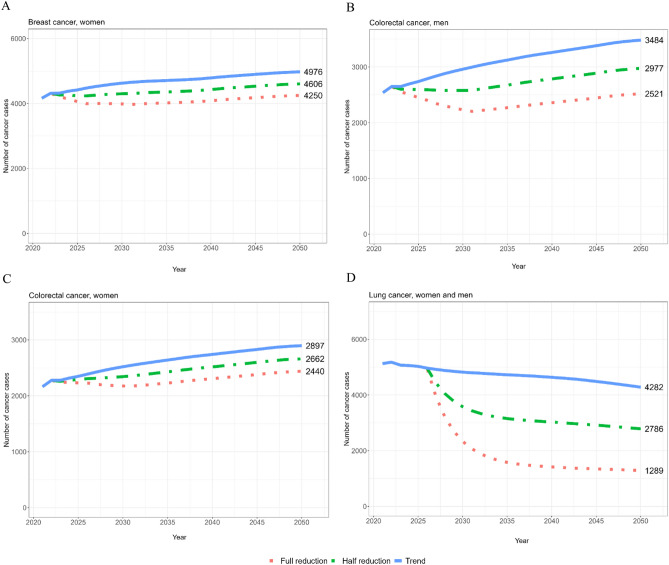
(**A**–**D**) Estimates of the number of new breast cancers in women (**A**), colorectal cancers in men (**B**) and women (**C**), and lung cancers (in men and women combined, (**D**) in 2050 in Denmark, showing the estimated trend with no intervention and the effects of instant 50% and 100% reductions in tobacco smoking (lung and colorectal cancer), overweight and obesity (colorectal and breast cancer), and alcohol consumption (colorectal and breast cancer) beginning in 2022.

In 2050, an increase of 25% (5775 total cases) in new prostate cancer cases was projected, along with a 19.3% (4976 total cases) increase in female postmenopausal breast cancer cases (Table 3, Fig. 1A).

Estimates for colorectal cancer in men and women showed increases of 38.5% (3484 total cases, Fig. 1B) and 33.4% (2897 total cases, Fig. 1C), respectively.

Male and female decreases were projected for lung cancer, with a total reduction of 17.7% (4282 total cases, Fig. 1D).

### Projected prevention scenarios

We expect the most significant reduction in new lung cancer cases in the year 2050, with a total possible decrease of 69.9% (2993 cases) with an instant elimination of smoking in men and women (Table 2 and Fig. 1D). Data were combined due to similarities in the trends. The second highest reduction was observed for colorectal cancer in men, with an estimated reduction of 27.6% (963 cases) in 2050 when tobacco smoking, overweight and obesity, and alcohol consumption were eliminated instantly (Table 2, Fig. 1B). Among women, the estimated reduction in new yearly colorectal cancer cases was lower, 15.8% (457 cases) (Table 2, Fig. 1C).

Breast cancer in postmenopausal women had an estimated reduction of 14.6%, equivalent to 726 cases in 2050, caused by an instant reduction in overweight and obesity, and alcohol consumption (Table 2, Fig. 1A).

Neither smoking, overweight and obesity, nor alcohol consumption increases the risk of prostate cancer, which is why no estimated reduction in new cancer cases in 2050 was observed in scenarios A or B (Table 2, Supplementary B).

Sensitivity analyses using the highest and lowest risk estimates from Table 1 showed a variation in the breast cancer estimate of ± 5.1%, ± 9.6%, and ± 9.8% in colorectal cancer in men and women, respectively. In lung cancer in men and women, the variation in the estimate was ± 8.35% (Supplementary B).

### Comparing prevention scenarios to present

When comparing the number of new cancer cases in 2021 to that in scenario B (100% instant elimination) in 2050 (Table 3), an increase in incident prostate cancer cases of 1,155 (25%) was seen (Supplementary A). This was equivalent to the trend in female postmenopausal breast cancer cases of 78 (1.9%) (Fig. 1 A), supplemented with a slight increase. Estimates for male and female colorectal cancer showed minor increases of 5 (0.2%) and 269 (12.4%) new cases, respectively (Fig. 1B, C). For lung cancer in men and women, a decrease of 3912 new cases, in total, was estimated to be -75.2% (Fig. 1D).

## Discussion

Our estimates show that with 100% prevention of smoking, overweight and obesity, and alcohol consumption beginning in 2022, the incident number of breast and colorectal cancer cases will remain at the same level in 2050, as the effects of demographic changes in population composition outweigh the prevention potential. Lung cancer cases will decrease by 75% compared to 2021 (Table 3). An increase of 25% in new prostate cancer cases was estimated since we could not point to any preventive factors. As the 100% reduction scenario is unattainable and hypothetical, this represents a conservative estimate of the unavoidable increase in future incident cancer cases, which must be emphasized in future health strategies.

From the estimated trend (with no intervention), we found the most significant increase in new cancer cases among prostate and colorectal cancer in 2050, with prostate cancer being the most common cancer. The latter finding aligns with data on future predictions from NORDCAN^45^ (Supplementary A). We found the greatest potential for prevention of lung cancer in men and women, with almost 70% (Table 2) of new cases being preventable by 2050 when tobacco smoking is eliminated, followed by colorectal cancer in men, and the lowest prevention potential was found in breast cancer in postmenopausal women.

As smoking is the leading cause of lung cancer and is currently responsible for at least three out of four incident cases in Denmark^39^, there is considerable potential for prevention. A significant effect on incident lung cancers is observed when smoking is reduced by 50% (Fig. 1D), which might be achievable in Denmark, combined with the decreasing trend. Smoking might influence the risk of developing prostate cancer, but the evidence is not clear. Neither alcohol nor overweight are proven to affect the risk^46^, and the most established risk factors for prostate cancer are nonmodifiable (gender, age, ethnicity, family history, and genetic variation)^47^. No prostate cancer cases could be prevented in 2050, but every sixth colorectal cancer in women could be prevented, and every fourth could be prevented in men.

Compared to Andersson et al.^17,19^, we reached similar estimates for preventable breast and colorectal cancer cases. The differences are mainly due to differences in the RR estimates. In our study, we added the differentiation of secondhand and former smokers, where Andersson et al. considered only current smokers. Regarding lung cancer, our estimate of preventable cases is comparable to that of a previous study by Andersen et al.^18^ but smaller than that of studies from Norway and the UK^48,49^. Primarily because our RR estimates are lower but also because we used the latest data on smoking prevalence, which is lower since the smoking prevalence has decreased in Denmark in the last 20 years and has been more than halved since 2000^50^.

Compared to other reports from Denmark from the Information Board for Prevention^51^, we found our estimates to have a higher number of preventable cases of breast, colorectal, and lung cancer. However, the report included data on preventable cancers in 2014 compared to our study, which makes estimates for the year 2050 and includes expected increases in cancer incidence.

From the sensitivity analyses, we found the biggest variations in colorectal cancer in men and women. This is the only cancer affected by all three risk factors. However, we saw relatively minor differences in the estimates.

## Strengths and limitations

Our study uses Danish data, but our data might be comparable to those of other Western countries with similar prevalence of risk factors and demographic profiles. A strength of our study design using the Prevent program is that similar analyses could be made in other Western countries, including more risk factors and cancer types. However, the calculations made in Prevent are based on the data input, and the results will depend on the data quality. The model should not be considered reliable for precise predictions of future cancer incidence but rather as a tool for evaluating the possible effects of theoretical prevention outcomes on disease incidence.

Another strength of our study is that we used the latest data on risk factor prevalence and highly reliable incidence rates from NORDCAN. In most cases, the RRs applied in our study were adjusted for confounders in the original studies.

Data on risk factor prevalence relies on survey data shown to underestimate, e.g., alcohol consumption^52^. In our study, this would cause an underestimation of preventable breast and colorectal cancer cases. Furthermore, we were not able to adjust for joint effects due to a lack of data on combined exposure to risk factors, e.g., the impact of alcohol consumption on colorectal cancer seems to be increased by obesity^53,54^. This could cause a slight overestimation of the number of non-preventable cancers. However, the prevalence of individuals exposed to multiple risk factors is expected to be a small fraction of the total population. Moreover, the major cancers addressed in our study have less clear evidence of joint effects.

On the other hand, due to simplicity, we did not account for the fact that prevention of one type of cancer, with an expected increase in survival time, could lead to the development of another type of cancer. In some individuals, this would increase the likelihood of opportunistic testing for slower-growing tumors, and in this circumstance, our presented number of non-preventable cancers could be underestimated.

Prevent assumes that the distribution of risk factors is equal in the population; therefore, we cannot consider differences in, e.g., socioeconomic status and modifiable risk factors and their relation to cancer risk, and it does not provide the possibility of confidence intervals.

The sensitivity analyses provide a picture of the uncertainty and current evidence of the associations between the risk factors and the risk of developing cancer. Since we have no estimates of uncertainty of the prevalence of the risk factors or the population predictions, we could not test the sensitivity of those variables.

We did not consider physical inactivity or red/processed meat, which are known to increase the risk of breast cancer (only physical inactivity) and colorectal cancer but instead associated with lower relative risk estimates, as shown by Tybjerg et al. Neither did we include hormone replacement as a risk factor for breast cancer; therefore, we were not able to adjust for these risk factors.

## Implications

This study provides estimates that new cancer cases increase regardless of conservative and unattainable scenarios of preventing modifiable risk factors. Smoking, overweight and obesity, and alcohol consumption are known to contribute to the development of cancer. Still, our study showed that even interventions eliminating all risk factors could merely achieve a status quo number of cancer cases.

Pressure on the health system and an increase in incident cancers are unavoidable in Denmark and probably also in other Western countries, as an increase in the elderly population primarily causes the rise in the number of new cancers. Moreover, improved cancer treatment has caused mortality rates to decrease in Denmark, and more cancer patients survive long enough to develop a second and third primary cancer. A newly published study showed that primary cancer related to smoking or alcohol consumption increases the risk of secondary cancer with the same etiology^55^.

The consequences for the health system will mean more cancer patients in the future, requiring increased expenses for diagnostics, treatment in the form of surgery, and medical interventions along with rehabilitation and follow-up.

## Conclusion

Preventing modifiable risk factors should lead the way to a healthier society and increase life expectancy. However, for the most common cancers, the number of new cancer cases per year will inevitably increase in the future, even with the implementation of comprehensive and conservative prevention strategies. The increase in the elderly population will drive it.

This should be acknowledged in future health planning strategies, and the expected increase in prevalence should lead to additional research on early detection and targeted screening.

### Supplementary Information

Supplementary Information 1.

Supplementary Information 2.

## Data Availability

Data is provided within the manuscript or supplementary information files. Updated prevalence data are publicly available at www.danskernessundhed.dk, cancer incidence data at www.nordcan.iarc.fr/en, and population projections at www.dst.dk.
