# Supplementary material for: Non-preventable cases of breast, prostate, lung, and colorectal cancer in 2050 in an elimination scenario of modifiable risk factors
Source: Sci Rep. 2024 Apr 13;14:8577. doi: 10.1038/s41598-024-59314-x (PMC11016117; doi:10.1038/s41598-024-59314-x)
Supplement: Supplementary file 2 — Supplementary Information 2. [file 41598_2024_59314_MOESM2_ESM.docx]

Supplementary B

# Results

*Sensitivity analyses*

Sensitivity analyses with 50% and 100% instant reduction in modifiable risk factors were made using the relative risk estimates’ lowest and highest 95% confidence interval (95% CI) shown in Table 1 in the original article.

Results are summarized in Tables S1-S2 and Figures S3-S6.

| Table S1 Non-preventablecancer cases in 2050 in Denmark predicted in numbers (#) and percentage with current trend (No intervention) and in scenarios of reduction of modifiable risk factors using the lowest 95% CI. | | | | | |
| --- | --- | --- | --- | --- | --- |
| Cancer type | No intervention | 50% instant reduction* | | 100% instant reduction§ | |
|  | # | Intervention # | Difference¤  # (%) | Intervention # | Difference¤  # (%) |
| Prostate | 5,775 | 5,775 | 0 (0) | 5,775 | 0 (0) |
| Breast (postmenopausal)^a^ | 4,962 | 4,718 | -244 (-4.9) | 4,481 | -481 (-9.7) |
| Colorectal, men^b^ | 3,507 | 3,176 | -331 (-9.4) | 2,865 | -642 (-18.3) |
| Colorectal, women^b^ | 2,896 | 2,796 | -100 (-3.5) | 2,698 | -198 (-6.8) |
| Lung, men^c^ | 2,106 | 1,447 | -659 (-31.3) | 789 | -1,317 (-62.5) |
| Lung, women^c^ | 2,205 | 1,556 | -649 (-29.4) | 907 | -1,298 (-58.9) |
| Lung, men and women^c^ | 4,311 | 3,003 | -1,308 (-30.3) | 1,696 | -2,615 (-60.7) |

Table S1.

*50% instant reduction in modifiable risk factors instantly from 2022.

§100% instant elimination in modifiable risk factors instantly from 2022.

¤Difference compared to no intervention scenario.

^a^ Reduction in overweight and obesity, and alcohol consumption.

^b^ Reduction in overweight and obesity, smoking, and alcohol consumption.

^c^ Reduction in smoking.

| Table S2 Non-preventable cancer cases in 2050 in Denmark predicted in numbers (#) and percentage with current trend (No intervention) and in scenarios of reduction of modifiable risk factors using the highest 95% CI. | | | | | |
| --- | --- | --- | --- | --- | --- |
| Cancer type | No intervention | 50% instant reduction* | | 100% instant reduction§ | |
|  | # | Intervention  # | Difference¤  # (%) | Intervention  # | Difference¤  # (%) |
| Prostate | 5,775 | 5,775 | 0 (0) | 5,775 | 0 (0) |
| Breast (postmenopausal)^a^ | 4,992 | 4,484 | -508 (-10.2) | 4,003 | -989 (-19.8) |
| Colorectal, men^b^ | 3,458 | 2,761 | -697 (-20.2) | 2,164 | -1,294 (-37.4) |
| Colorectal, women^b^ | 2,897 | 2,496 | -401 (-13.8) | 2,133 | -764 (-26.4) |
| Lung, men^c^ | 2,071 | 1,255 | -816 (-39.4) | 439 | -1,632 (-78.8) |
| Lung, women^c^ | 2,195 | 1,360 | -835 (-38.0) | 525 | -1,670 (-76.1) |
| Lung, combined^c^ | 4,266 | 2,615 | -1,651 (-38.7) | 964 | -3,302 (-77.4) |

Table S2.

*50% instant reduction in modifiable risk factors instantly from 2022.

§100% instant elimination in modifiable risk factors instantly from 2022.

¤Difference compared to no intervention scenario.

^a^ Reduction in overweight and obesity, and alcohol consumption.

^b^ Reduction in overweight and obesity, smoking, and alcohol consumption.

^c^ Reduction in smoking.

S3


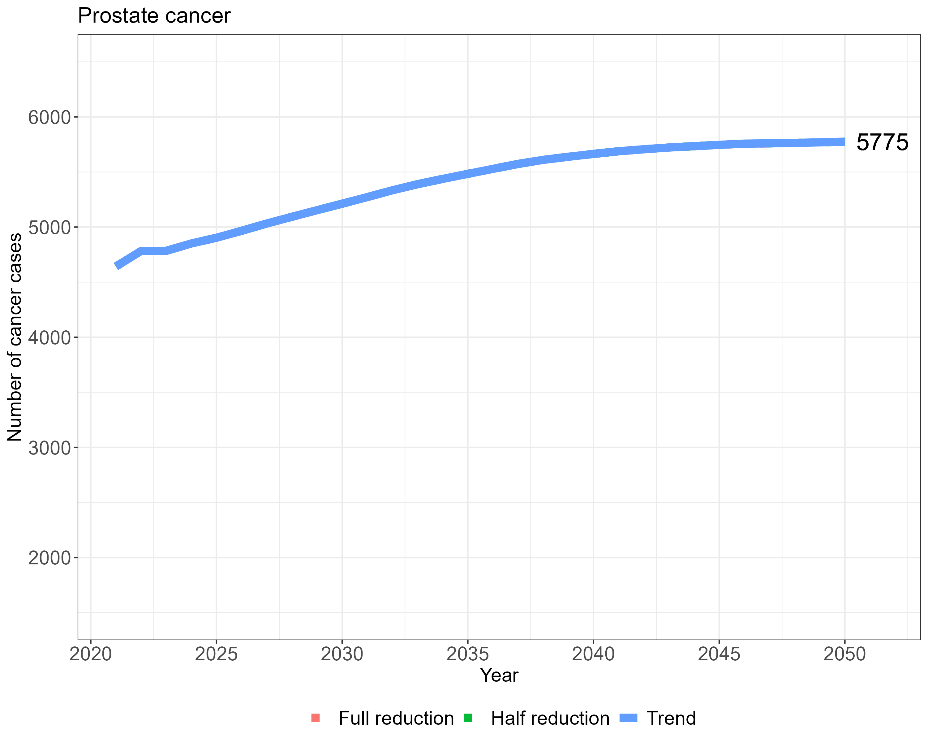


Figure S3.

Estimate for the number of new prostate cancer cases in 2050 in Denmark showing the estimated trend and no effects of modification in major risk factors.

S4a

S4b


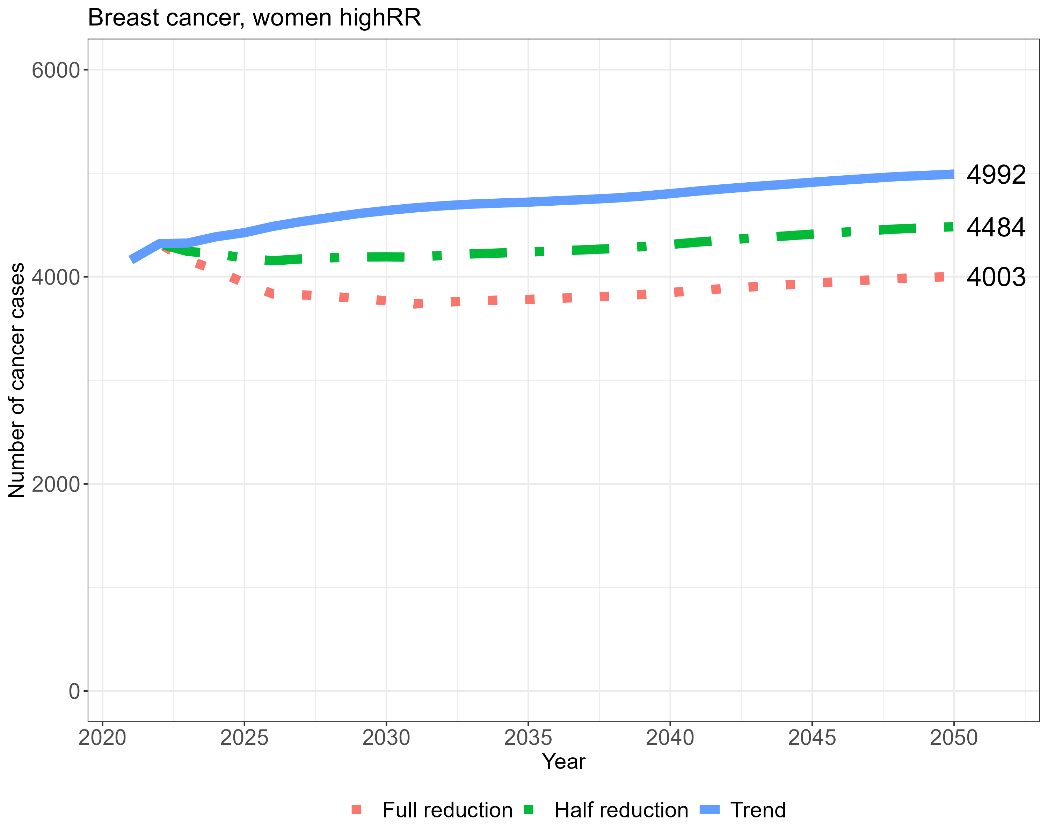

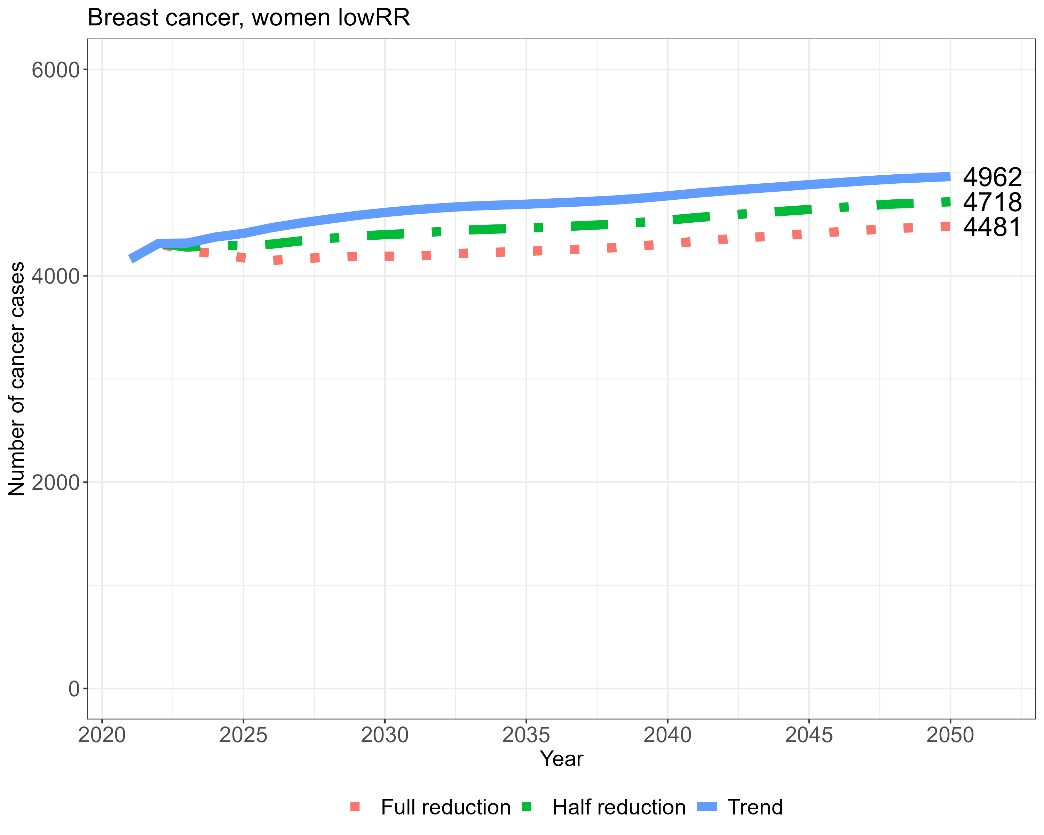


Figure S4a-b.

Sensitivity analysis of the estimates for the number of new breast cancer cases in postmenopausal women in 2050 in Denmark, using the highest and lowest relative risk estimates from the 95% confidence interval from Table 1, showing the estimated trend with no intervention and the effects of an instant 50% and 100% reduction in overweight and obesity and alcohol consumption beginning in 2022.

S5b

S5a


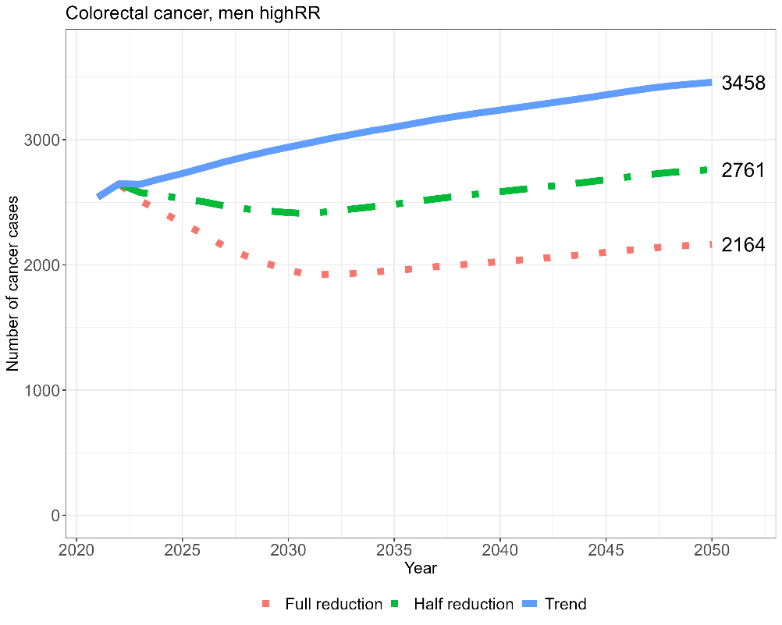

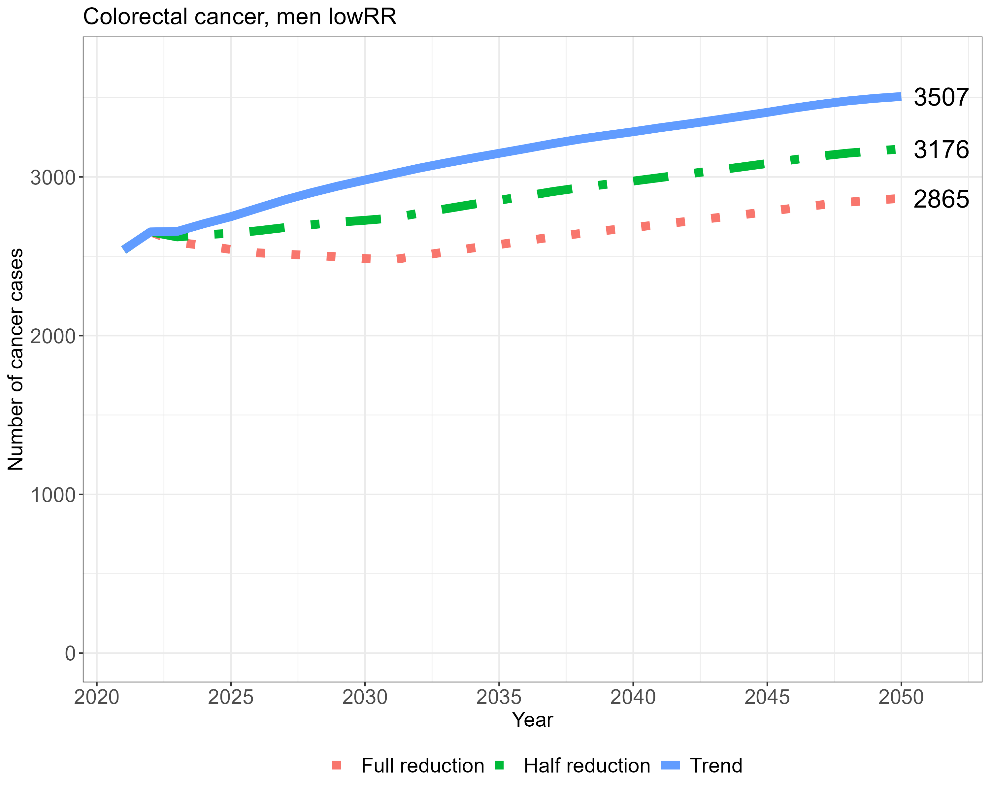


Figure S5a-d.

Sensitivity analyses of the

estimates for the number of

new colorectal cancer cases

in men and women in 2050

in Denmark, using the highest

and lowest relative risk

estimates from the 95%

confidence interval from

Table 1 shows the estimated

trend with no intervention and

the effects of an instant 50%

and a 100% reduction in

overweight and obesity,

alcohol consumption,

and tobacco smoking

beginning in 2022.

S5c

S5d


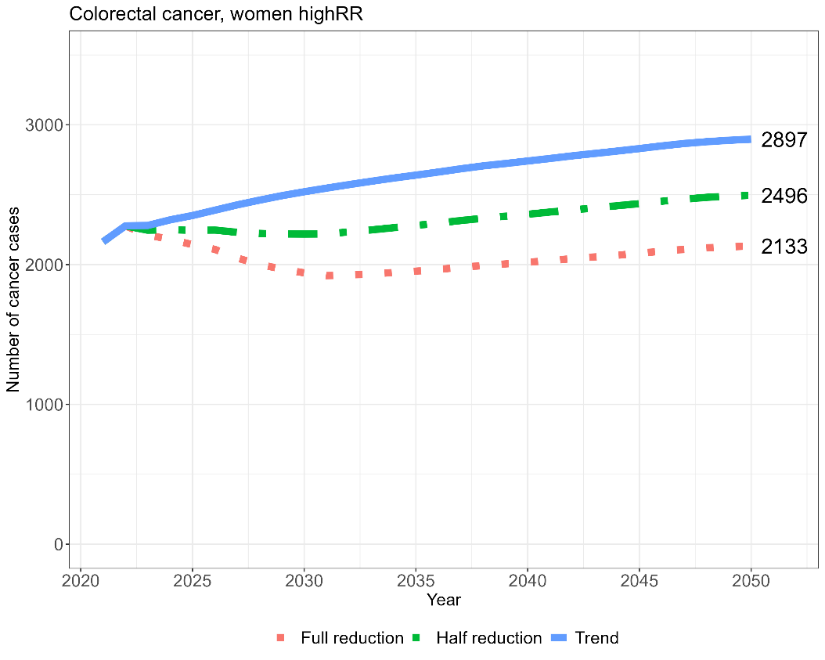

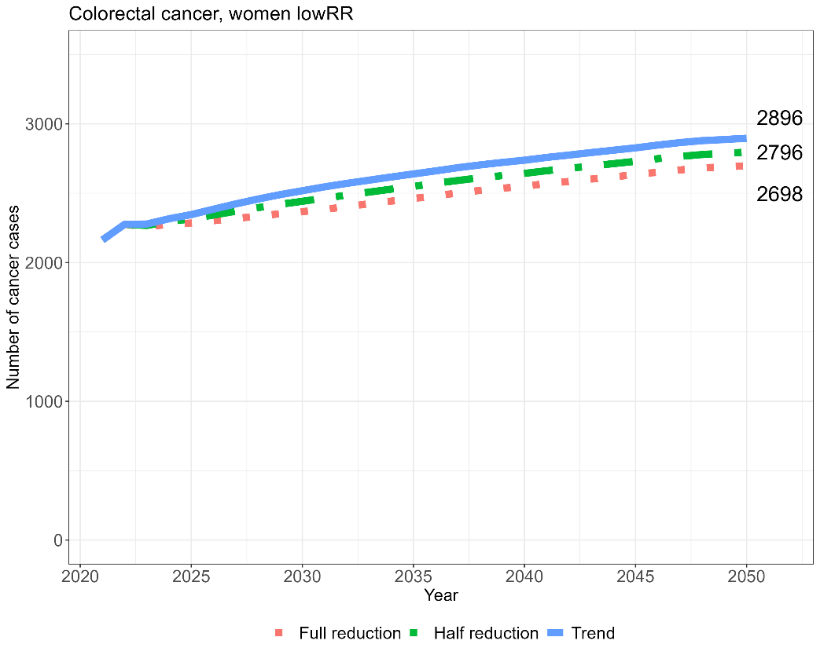


S6a


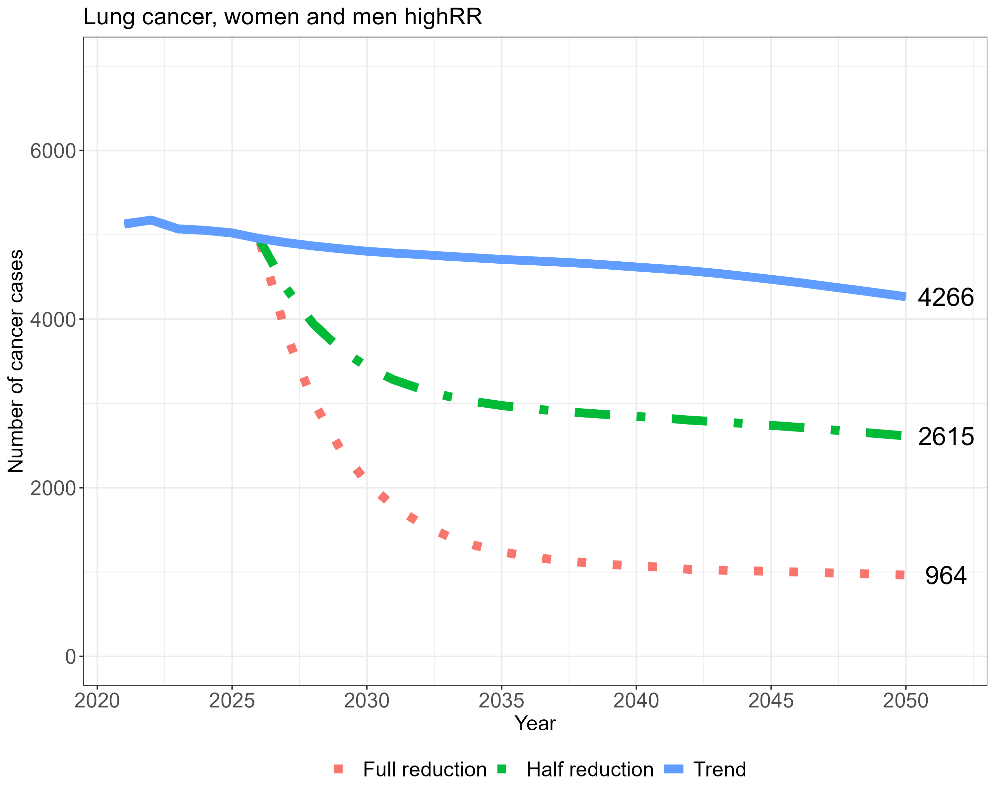

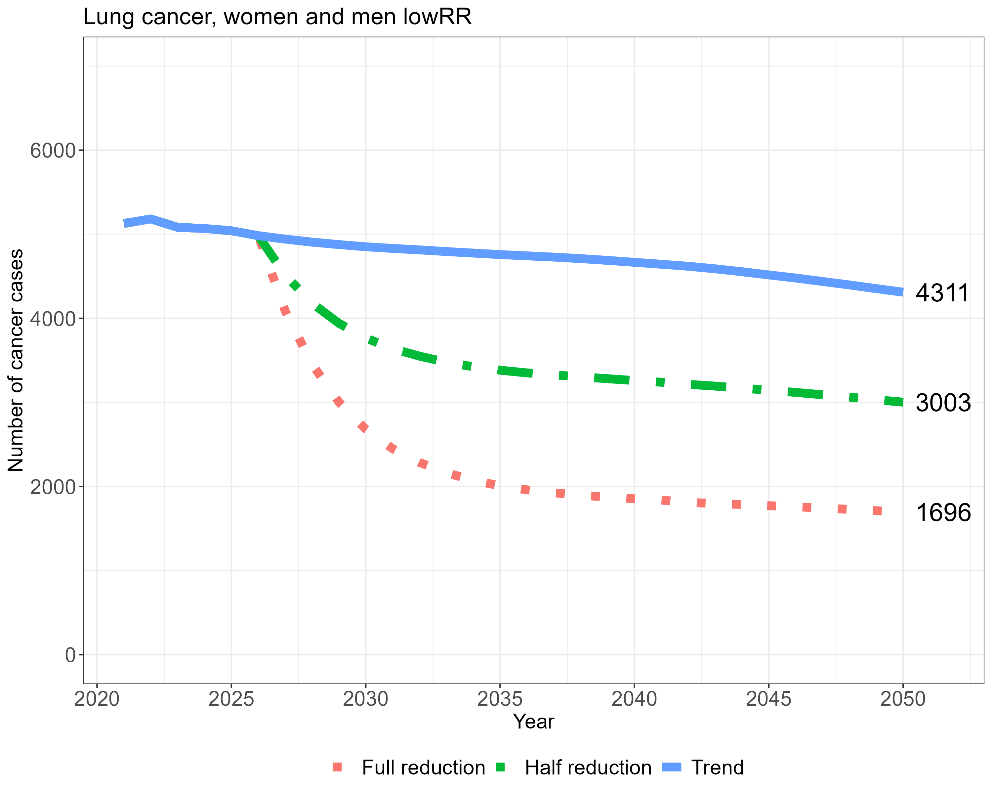


S6b

Figure S6a-b.

Sensitivity analysis of the estimates for the number of new lung cancer cases in women and men in 2050 in Denmark, using the highest and lowest relative risk estimate from the 95% confidence interval from Table 1, showing the estimated trend with no intervention and the effects of an instant 50% and 100% reduction in tobacco smoking beginning in 2022.
